# Application of structural equation models to construct genetic networks using differentially expressed genes and single-nucleotide polymorphisms

**DOI:** 10.1186/1753-6561-1-s1-s76

**Published:** 2007-12-18

**Authors:** Seungmook Lee, Mina Jhun, Eun-Kyung Lee, Taesung Park

**Affiliations:** 1 Department of Statistics, Seoul National University, San 56-1, Sillim-dong, Gwanak-gu, Seoul 151-742, Korea; 2 Interdisciplinary Program in Bioinformatics, Seoul National University, San 56-1, Sillim-dong, Gwanak-gu, Seoul 151-742, Korea

## Abstract

Understanding the genetic basis of human variation is an important goal of biomedical research. In this study, we used structural equation models (SEMs) to construct genetic networks to model how specific single-nucleotide polymorphisms (SNPs) from two genes known to cause acute myeloid leukemia (AML) by somatic mutation, runt-related transcription factor 1 (*RUNX1*) and ets variant gene 6 (*ETV6*), affect expression levels of other genes and how *RUNX1 *and *ETV6 *are related to each other. The SEM approach allows us to compare several candidate models from which an explanatory genetic network can be constructed.

## Background

To understand the genetic basis of complex traits, it is often useful to examine intermediate gene expression traits. It is known that many diseases occur because of changes in either patterns or levels of gene expression [1,2]. There are also known cases in which differences in gene expression due to genotype are associated with phenotypic variation [3-7]. Thus, the analysis of gene expression may lead to a better understanding of the genetic basis of complex traits [3]. The study of gene expression variation, both within and between species, is currently an active area of research [8-10].

We propose an approach based on structural equation models (SEMs) [11] to construct a genetic network that can provide information about the genetic basis of expression variation in humans. SEMs were originally developed in the early 1970s in the field of social science to fit models with unobserved variables. A key feature of the SEM approach is that it allows one to compare candidate models.

In this study, we used gene expression data and SNP data provided by the Genetic Analysis Workshop 15 (GAW15). The study subjects were selected from 14, three-generation Utah families with ancestry from northern and western Europe (Utah Centre d'Etude du Polymorphisme Humain, or CEPH). Each family consisted of 14 individuals (4 grandparents, 2 parents, and 8 offspring), except for two families that had only 7 offspring (so only 13 individuals). In these families, expression levels of 8500 genes in lymphoblastoid cells were initially measured. Morley et al. [12] found that 3554 genes out of these 8500 genes showed greater variations among individuals than between replicate determinations in the same individual. Expression levels of these 3554 genes were provided to GAW15. The GAW15 genotype data contained 2882 autosomal and X-linked single-nucleotide polymorphisms (SNPs) for each member of the 14 CEPH Utah families. The genotypes were generated by the SNP Consortium .

We are interested in acute myeloid leukemia (AML). In this study, we focused on two known AML-related genes, runt-related transcription factor 1 (*RUNX1*) and ets variant gene 6 (*ETV6*). Although expression levels for these two genes were not included in the GAW15 data set, we used two-way analysis of variance (ANOVA) models to find other genes that were differentially expressed depending on the genotype for SNPs within *RUNX1 *and *ETV6*. Using genes selected from the ANOVA, we fitted SEMs and constructed genetic networks.

## Methods

### Selection of differentially expressed genes from SNPs

First, we used dbSNP (Build 126) and the Online Mendelian Inheritance in Man (OMIM) FTP site of the National Center for Biotechnology Information (NCBI) to select SNPs within *RUNX1 *and *ETV6*. Next, we used the following model to fit two-way ANOVA models to find differentially expressed genes for each selected SNP:

*y*_*ijk *_= *μ *+ *FAMID*_*i *_+ *SNP*_*j *_+ FAMID*SNP_*ij *_+ *ε*_*ijk*_,

where *y*_*ijk *_is the expression level of the gene in the *k*^th ^individual with SNP genotype *j *from family *i. FAMID *and *SNP *represent the family and SNP effects, respectively, and *ε*_*ijk *_is a random error term. A gene was selected to be part of the SEM analysis if the fit of the full model was significantly better (*p *< 0.05) than the model that included only overall mean and family.

### Structural equation models for genetic networks

SEMs are comprehensive statistical models that allow us to test relations among observed and latent variables; in this context, we used them to construct a network of differentially expressed genes for specific polymorphisms. Latent variables are unobserved variables that are implied by the covariance among two or more indicators. The expression levels for *RUNX1 *and *ETV6 *were not included in the data provided to GAW15. Because of this, and the way we selected our observed variables, we believe it is likely that the latent variables represent the level of expression of these two genes.

**Figure 1:**
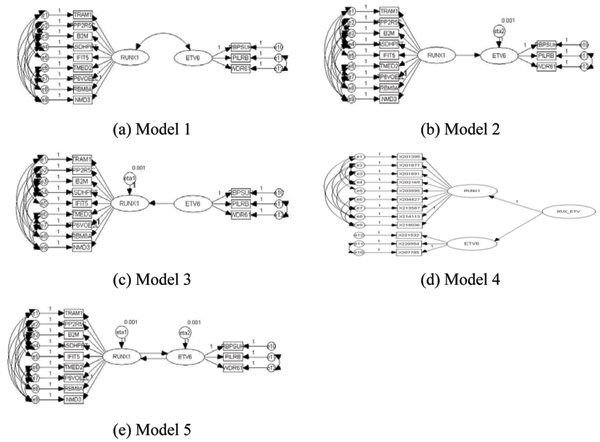
Diagram of SEMs for constructing genetic structures.

Path diagrams were used to describe genetic networks in a graphical manner (Figure 1). There are two types of variables used in path diagrams: observed variables (represented by rectangles) and latent variables (represented by ovals). The relationships defined in SEMs consist of two parts: relationships among the latent variables and relationships between the latent variables and the observed variables. The SEMs for our genetic networks were defined as follows:

*y *= Λ_*y*_*η *+ *ε*,

*η *= *Bη *+ *ς*,

where *y *is a vector representing the observed variables (gene expression levels); *η *is a vector of the latent variables (*RUNX1 *and *ETV6 *expression); Λ_*y *_is a matrix representing the true relationships between the gene expression levels and the gene functions; and *B *is a matrix representing the true the relationships among the latent variables. Random errors in the equations are represented by *ε *and *ς*. To fit this model, we used AMOS, a SEM software solution provided by SPSS .

## Results

### SNP selection

From the 2882 SNPs provided in the GAW15 data set, we identified six SNPs located on *RUNX1 *and *ETV6 *on the basis of chromosomal location. The *RUNX1 *SNPs are rs882776, rs933131, and rs1892687 and the *ETV6 *SNPs are rs1894307, rs1573612, and rs916041.

Although we selected SNPs based solely on physical position, we expect that *RUNX1 *and *ETV6 *are co-regulated and are involved in the regulation of additional genes. We constructed the SEMs after identifying genes apparently regulated by these six SNPs.

### Construction of a genetic network for differentially expressed genes using SEMs

**Figure 2:**
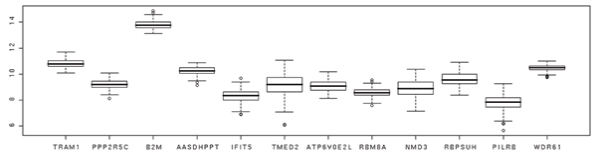
Box plot of selected gene expressions.

From our ANOVA analyses, we identified nine differentially expressed genes associated with the RUNX1 SNPs: translocation associated membrane protein 1 (*TRAM1*); gamma isoform (*PPP2R5C*); beta-2-microglobulin (*B2M*); aminoadipate-semialdehyde dehydrogenase-phosphopantetheinyl transferase (*AASDHPPT*); interferon-induced protein with tetratricopeptide repeats 5 (*IFIT5*); transmembrane emp24 domain trafficking protein 2 (*TMED2*); ATPase, H+ transporting V0 subunit E isoform 2-like (rat) (*ATP6V0E2L*); RNA binding motif protein 8A (*RBM8A*); and *NMD3 *homolog (*NMD3*). Similarly, we identified three genes with expression correlated to the *ETV6 *SNPs: recombining binding protein suppressor of hairless (*RBPSUH*); paired immunoglobin-like type 2 receptor beta (*PILRB*); and WD repeat domain 61 (*WDR61*). Figure 2 shows the box plots of expression levels for these 12 selected genes.

First, we applied confirmatory factor analysis [11] to validate the use of these 12 differentially expressed genes in our SEM. Then, we added the relation between the latent variables and fitted the general SEMs.

To construct the genetic network, we treated *RUNX1 *and *ETV6 *as latent variables and all genes with expression values as observed variables. We considered SEMs where both *RUNX1 *and *ETV6 *could control any of the 12 expressed genes.

We fitted various SEMs to the data, as shown in Figure 1. Model 1 assumes that there is a correlation between the two latent variables and Models 2 and 3 assume that there is a one-sided causal relationship between them. Model 4 is a second-order factor model in which the latent variables directly influencing the observed variables may be influenced by other latent variables that need not have direct effects on the observed variables [11]. Finally, Model 5 assumes that there are mutual causal relationships between the two latent variables.

Table 1 shows goodness-of-fit measures for the five models. The goodness-of-fit index (GFI) measures the relative differences between data and estimated values obtained from a model, while the adjusted GFI (AGFI) adjusts the GFI according to the degrees of freedom. If these two measures are close to 1, we conclude that the model fits the data well. Smaller root mean square residuals (RMRs) indicate better-fitting models. The Akaike information criterion (AIC) is a well-known measure that can be used for the model comparison. A smaller AIC indicates a better-fitting model.

**Table 1:** Goodness-of-fit measures for SEMs

Measure	*χ*^2 ^statistic	GFI	AGFI	RMR	AIC
Model 1	167.902	0.891	0.757	0.023	253.902
Model 1 without 4 NS genes	58.748 (df = 26)	0.935	0.765	0.013	110.748
Model 2	194.660	0.876	0.731	0.031	278.660
Model 3	194.550	0.876	0.732	0.031	278.550
Model 4	229.415	0.849	0.690	0.021	309.415
Model 5	196.301	0.873	0.724	0.030	280.301

For Models 1–5, we tested, using the modification index (MI), many modified SEMs with different covariance structures for the error terms. The MI measures how much the chi-square statistic is expected to decrease if a particular constrained parameter is set free and the model is re-estimated [11]. In our model fitting, we first evaluated all pair-wise error connections and chose the connection with the largest MI. Given the first connection, we then chose the best remaining possible connection, and so forth. We continued until the improvement in fit was small.

Figure 1 illustrates the models that best fit the covariance structure for the error terms. Table 1 summarizes goodness of fit measures for these models. Model 1 provided the best fit, yielding the largest GFI and AGFI and the smallest AIC. For Models 2, 3, and 5, the estimates of the error variances were negative, indicating that these models represent the so-called "Heywood cases" [11]. Model 4 provided the best result in terms of the RMR but the worst result in terms of other goodness-of-fit measures such as the AIC. Because the AIC and RMR are different measures of goodness-of-fit, they might provide inconsistent results. One advantage of the AIC is that it considers the number of parameters in the model, while the RMR does not. Thus, we selected Model 1 as the best model in terms of the AIC.

The fitted results of Model 1 are summarized in Table 2; the results show that the latent variables we believe represent *RUNX1 *and *ETV6 *expression are closely related to each other. The parameter relating these latent variables (Table 2) estimates the covariance between *RUNX1 *and *ETV6*.

**Table 2:** Parameter estimates for Model 1

			Including all 12 measured genes				Excluding the 4 NS genes			
				
			Estimate	SE	*t*-value	*p*-value	Estimate	SE	*t*-value	*p*-value
RUNX1	→	NMD3	1				1			
		RBM8A	-0.294	0.058	-5.029	<0.001	-0.269	0.062	-4.300	<0.001
		ATP6V0E2L	-0.683	0.053	-13.007	<0.001	-0.674	0.054	-12.581	<0.001
		TMED2	0.19	0.118	1.603	0.109				
		IFIT5	0.095	0.061	1.572	0.116				
		AASDHPPT	0.061	0.048	1.277	0.202				
		B2M	-0.221	0.05	-4.382	<0.001	-0.214	0.049	-4.334	<0.001
		PPP2R5C	0.572	0.059	9.702	<0.001	0.563	0.058	9.659	<0.001
		TRAM1	0.045	0.041	1.087	0.277				
ETV6	→	RBPSUH	1				1			
		PILRB	-0.368	0.095	-3.866	<0.001	-0.390	0.1	-3.911	<0.001
		WDR61	-0.123	0.037	-3.320	<0.001	-0.092	0.039	-2.389	0.017
ETV6	↔	RUNX1	0.213	0.028	7.543	<0.001	0.221	0.03	7.351	<0.001

Of the 12 genes with measured expression, all three that were differentially expressed for *ETV6 *showed significant connection to the latent variable representing *ETV6*. Among the nine differentially expressed genes for *RUNX1*, four – *TMED2*, *IFIT5*, *AASDHPPT*, and *TRAM1 *– did not show significant control by the latent variable *RUNX1*. When we excluded these nonsignificant genes, the goodness-of-fit measures improved compared to the full Model 1 (Table 1) while the parameter estimates and significance (Table 2) remained similar to those of the full model.

## Conclusion

In this paper, we proposed the use of SEMs to construct genetic network models. We demonstrated this method using two genes, *RUNX1 *and *ETV6*, which are well known to cause AML by somatic mutation [13]. We used SNPs in these genes to select the differentially expressed genes related to these SNPs to use as the observed variables for our SEMs. The best-fitting model indicated that, even though we did not have direct measurements of *RUNX1 *and *ETV6 *expression, they are likely to be highly correlated with each other.

In summary, SEMs allow us to compare candidate models and to construct a genetic network from the best fitting model. However, the SEM approach has several limitations, such as 1) as the number of genes increases, the number of parameters in the model can increase exponentially; 2) if there are many genes, it is difficult to determine the causality relationship among the genes; 3) it can be difficult to interpret the relationship embedded in SEMs. For these reasons, in order to construct the genetic network efficiently, it is very useful to find a few genes that are correlated with other genes in the proposed network. Another limitation of the SEM approach is that it requires the selection of an initial model that is updated to construct the final SEM. As described above, this process can require many iterations to determine an appropriate error structure. In spite of these limitations, we believe that our application of SEMs to the GAW15 data demonstrates that the SEM approach can be a useful and efficient method for constructing informative genetic networks.

## Competing interests

The author(s) declare that they have no competing interests.
